# Conflicting discourses of church youths on masculinity and sexuality in the context of HIV in Kinshasa, Democratic Republic of Congo

**DOI:** 10.1080/17290376.2014.930695

**Published:** 2014-07-07

**Authors:** Hendrew Lusey, Miguel San Sebastian, Monica Christianson, Lars Dahlgren, Kerstin E. Edin

**Affiliations:** ^a^ (RN, MPH, PhD student) is the Central Africa Regional Coordinator of the Ecumenical HIV and AIDS Initiative in Africa (EHAIA), World Council of Churches, Kinshasa, Democratic Republic of Congo; ^b^ Department of Nursing, Umeå University, Umeå, Sweden; ^c^ Department of Public Health and Clinical Medicine, Epidemiology and Global Health, Umeå University, Umeå, Sweden; ^d^ (MD, PhD) is associate Professor at the Department of Public Health and Clinical Medicine, Umeå University, Umeå, Sweden; ^e^ (PhD, MPH, RNM) is Senior Lecturer at the Department of Nursing, Umeå University, Umeå, Sweden; ^f^ (PhD) is Professor Emeritus at the Department of Sociology, Umeå University, Umeå, Sweden; ^g^ (PhD, MPH, RNM) is Senior Lecturer both at the Department of Nursing, Umeå University and at the Deparment of Public Health and Clinical Medicine, Umeå Center for Global Health Research, Umeå University, Sweden

**Keywords:** masculinity, sexuality, young churchgoers, HIV prevention, gender equality, DR Congo, masculinité, sexualité, jeunes chrétiens, prévention du VIH, égalité du genre, RD Congo

## Abstract

Masculinity studies are fairly new and young churchgoers are an under-researched group in the current Congolese church context. In response to this knowledge gap, this paper attempts to explore discourses of young churchgoers from deprived areas of Kinshasa regarding masculinity and sexuality in the era of HIV. A series of 16 semi-structured interviews were conducted with unmarried young churchgoers from the Salvation Army, Protestant and Revival churches. The interviews were tape-recorded, transcribed verbatim and analysed using discourse analysis. Five main discourses emerged: ‘we are aware of the church message on sex’, ‘young men need sex’, ‘young women need money’, ‘to use or not to use condoms’ and ‘we trust in the church message’. Although all informants knew and heard church messages against premarital sex, many of them were sexually active. The perception was that young men were engaged in sexual activities with multiple partners as a result of sexual motivations surrounding masculinity and sexual potency, while young women sought multiple partners through transactional and intergenerational sex for economic reasons. These sexual practices of young people conflicted with church messages on sexual abstinence and faithfulness. However, a small number of participants challenged current gender norms and suggested alternative ways of being a man or a woman. To elucidate these alternatives, we suggest that church youths and church leaders might take concrete actions to deconstruct misconceptions about being men. In this way, they can possibly enhance a frank and fruitful dialogue on sex, sexuality and gender to promote positive masculinities and constructive partnerships to prevent HIV.

## Résumé

Dans le contexte actuel des églises Congolaises, les études relatives à la masculinité sont presque récentes et les jeunes chrétiens constituent un groupe d'individus qui ne font pas l'objet des recherches scientifiques approfondies. En réponse à cette insuffisance de connaissances dans le contexte à VIH, le présent article tente d'explorer les discours relatifs à la masculinité et à la sexualité des jeunes chrétiens issus des quartiers défavorisés de Kinshasa. Une série de 16 interviews semi-structurées ont été menés auprès des jeunes chrétiens célibataires appartenant à l'Armée du Salut, aux églises Protestantes et à celles du Réveil du Congo. Les interviews ont été enregistrées et analysées en utilisant la méthode du discours. Cinq discours ont émergé notamment: « Nous connaissons le message des églises au sujet du sexe », « les garçons ont besoin des rapports sexuels », « les filles ont besoin d'argent », « faudrait-il utiliser ou ne pas utiliser les condoms » et « nous croyons dans le message des églises ». Alors que tous les participants connaissaient le message des églises qui interdisent les rapports sexuels prémaritaux, beaucoup d'entre eux étaient déjà sexuellement actifs. Les garçons ont été perçus comme des personnes qui ont des rapports sexuels avec plusieurs partenaires concomitants pour prouver leur masculinité et leur puissance sexuelle. Les filles chercheraient à avoir des rapports sexuels mercantiles et intergénérationnels avec des partenaires multiples à des fins économiques. Ces pratiques sexuelles des jeunes s'opposent aux discours des églises qui promeuvent l'abstinence sexuelle et la fidélité. Cependant, quelques participants ont remis en cause les normes courantes du genre et ont suggéré des alternatives en ce qui concerne l'identité des hommes et des femmes. Pour les élucider, nous proposons que les jeunes chrétiens et les leaders des églises puissent mener des actions concrètes dans le but de déconstruire les conceptions erronées de ce que veut dire être homme. Ce faisant, ils peuvent probablement maximiser les chances d'un dialogue franc et productif en ce qui concerne le sexe, la sexualité et le genre afin de promouvoir la masculinité positive et le partenariat constructif, susceptibles de prévenir l'infection à VIH.

## Introduction

The Democratic Republic of Congo (DRC) recognised HIV in 1983 and the current available HIV prevalence range from 1% to 7% in the general population. Among young people aged 15–24, the HIV prevalence is estimated to be around 4% (Rapport National UNGASS 2010); the same report indicates that HIV is the leading cause of both morbidity and mortality for Congolese aged 20–40 years. These rates could be uncertain due to the armed conflict, which has made it difficult to collect accurate data on HIV. This conflict, which is still ongoing, particularly in the eastern region, has taken more than 5.4 million lives over the last decade (Leaning, Bartels & Mowafi 2009). As a result, most of the health and basic infrastructure has deteriorated, while the conflict is known to have generated many of the conditions that increase the spread of HIV.

Currently, nearly 1.2 million Congolese are living with HIV, and among them approximately 300,000 are eligible to start antiretroviral therapy (ART) (USAID/Democratic Republic of the Congo 2010). However, the same USAID report stressed that despite the provision of funds from external partners, ART has only been planned for an estimated 67,000 people due to resource-related constraints under the current political instability in DRC. While ART prolongs and improves life, HIV prevention should continue to play the most important role. Thus, targeting youths lies at the heart of prevention that could save lives at a low cost in impoverished communities.

As in other sub-Saharan African countries, the HIV epidemic in DRC is mainly transmitted through unprotected sex (UNAIDS 2003). Studies have shown that poor and sexually active girls are vulnerable to HIV because they lack agency to negotiate safer sex not only with older men (Ghosh & Kalipeni 2005), but also, in general, when they exchange sex for money (Bhana & Pattman 2011). In order to prevent HIV, many interventions empower women and girls to protect themselves (Ricardo, Nascimento, Fonseca & Segundo 2010). Yet, female empowerment alone is insufficient to change young men's behaviours that are partly fuelling the HIV epidemic (Sathiparsad, Taylor & De Vries 2010).

According to the national Demographic and Health Survey (Ministry of Plan 2007), young Congolese men aged 15–24 had higher rates of HIV infection than young women of the same age. However, after the age of 25, women have higher rates of HIV than men. Young men engaging in early sexual debuts with multiple and concurrent partners (Nshindano & Maharaj 2008) and those living with HIV are more likely to infect many female partners. Hence, young men are perceived as key transmitters of HIV (Macia, Maharaj & Gresh 2011). However, there are also young men who protect themselves and their partners from HIV by being abstinent, monogamous and/or consistently using a condom (UNAIDS/01.24E 2001). As a result, there is compelling evidence that well-designed and effective public health programmes that work with men and boys can lead to positive changes in their attitudes towards sex and gender relations (Barker, Ricardo, Nascimento, Olukoya & Santos 2010).

In a review, Marston and King (2006) stated that a focus on 15–24-year olds can make a real impact on HIV, given the high risk of infection in this age group. In some African countries, sexual behaviours have been positively influenced in these young people (UNAIDS 2010). As a result, new infections are currently declining in 13 African countries among young people due to the adoption of sexual abstinence, reduction in the number of partners and safer sexual behaviours (Halperin, Mugurungi, Hallett, Muchini, Campbell, Magure, *et al*., 2011). Some of these strategies are in line with HIV preventive measures also promoted by churches.

The population of the DRC comprises a diversity of religious affiliations including Catholic (31%), Protestant (30%), other Christian churches (34%), Muslims (2%) and the remaining 3%, who are members of indigenous and syncretic religions (Macro International 2007). As such, religion plays an important role in the daily lives of most people in DR Congo, especially for those living with HIV, as they can find guidance and support from their churches (Maman, Cathcart, Burkhardt, Omba, & Behets 2009). Given the three decades of state mismanagement by the dictatorial regime, coupled with a decade of recurrent conflicts, the government's capacity in the provision of health care is severely limited. As a result, nearly 70% of health services are provided by churches and church-related institutions (Haddad, Olivier & De Gruchy 2008).

In the early days of the epidemic, however, many churches were inactive in responding to HIV because the leaders of certain churches saw the disease as God's punishment towards immoral people (Cordaid 2009). Consequently, some church leaders have kept silent on HIV since HIV is mainly transmitted through unprotected sex with an infected partner, particularly during premarital and extramarital sexes, which in churches have been considered to be associated with sin (Smith 2003). Although some Congolese churches are still working on HIV in fragmented ways, their commitment to collectively responding to HIV has improved in recent years.

In collaboration with the National AIDS Council, the Ecumenical HIV and AIDS Initiative in Africa, UNAIDS and other partners, mainline churches and the Muslim community have established an HIV interfaith platform in Kinshasa. In line with their doctrines, they emphasise sexual abstinence for the unmarried and monogamy for the married, while they recommend condom use only for couples where one partner is HIV positive. Churches have received substantial criticism related to HIV prevention from HIV activists and donor groups. For instance, some churches’ opposition to condom use (Bosmans, Cikuru, Claeys, & Temmerman 2006) and their dissemination of inaccurate HIV information to youths have been reported (Eriksson, Lindmark, Axiemo, Haddad & Ahlberg 2013). In spite of these shortcomings, several churches have compassionately pioneered HIV work (Vitillo 2009). A study in South Africa has shown the positive impact of an HIV church programme on prevention among youth groups (Mash & Mash 2012).

Masculinity studies in churches are fairly new (Chitando & Chirongoma 2012) and young churchgoers are an under-researched group (Nweneka 2007). While some church youths resist pressure for premarital sex, others adopt risky sexual behaviours (Mash, Kareiti & Marsh 2006). Many churches fail to challenge norms of gender inequality and reinforce them through selective reading of the Bible (Marshall & Taylor 2006). To the best of our knowledge, this is the first study which has been done on masculinity and sexuality in the Congolese church context. In response to the knowledge gap, this paper seeks to explore discourses related to masculinities and sexuality in the context of HIV among young male and female churchgoers in Kinshasa, DRC. The results of the study are expected to be useful when planning church HIV prevention activities among young men and women.

## Ethical approval

Ethical approval was obtained from the Kinshasa School of Public Health. The first author sought permission from church leaders and written informed consent was obtained from all interview participants except those who were below the age of 18, where in accordance to the Congolese law, permission was also requested from parents (Constitution de la République Démocratique du Congo 2006). Informants were assured voluntary participation, anonymity and non-judgmental attitudes during the interviews.

## Method

### Conceptual framework

Given the interest to understand men and masculinity in recent years, research has found that there are multiple ways of being a man. In this regard, Connell (1995) has suggested hegemonic, subordinated, complicit and marginalised masculinities, which are interrelated in various ways. Focusing on HIV in sub-Saharan Africa, Barker and Ricardo (2005) have examined the ways young men perceive their masculinities and found that masculinities are dynamic, socially constructed and vary across time, historical and local settings and may be influenced by religious, global, regional, and local practices (Spronk 2014).

In the field of sexuality, men are expected to be ready for sex at any time, to view sex as recreational, to see penetration as ‘real sex’ and to have sex with multiple partners (Mah & Halperin 2010). Men may abuse alcohol, which might impair their reflection in sexual encounters (Pithey & Parry 2009). In addition, men pay little attention for their health and are less likely to seek health care (Courtenay 2000). Contrary to these traditional norms of masculinities, which may put men themselves and their partners at increased risk of HIV, there is compelling evidence that men can and do change as a result of programme interventions (Barker, Nascimento, Segundo & Pulwerwitz 2004).

There is a growing consensus that discourse, the language used, is collectively and relationally constructed through people's daily interactions, and lies at the centre of understanding gender (Jorgensen & Philips 2002). Clark (2005) has identified discourses on different levels which implies that they can be constructed from significant others in the family, local neighbourhood as well as from others in the society. Discourses on masculinities are often complex and contradictory (Gibbs, Sikweyiya & Jewkes 2014), and change depending on social interactions, time and place. In this way, men are free to (re)construct male identities in their own ways such as in gender relations and sexual activities (Edley 2001). However, changes of masculinity identities require compromises and are therefore restricted because the negotiation includes power (Kimmel 1987; Spronk 2014). Hence, masculinity should be seen in terms of accomplishment rather than as a natural fact (Connell 1995). Consequently, the analysis of gender norms requires openness since discourses of masculinities are fluid (Connell 2008).

### Study setting

The study was carried out in the slum communities of Camp Luka, Bumbu and Masina in Kinshasa, the capital city of the DRC.

### Recruitment of participants

To be recruited to the study, participants had to be unmarried, attend church services and agree to be interviewed. While a few participants were recruited by their church leaders in one church, the first author selected informants to create a varied sample in terms of education and church backgrounds. In order to gain a deeper understanding of how masculinity is socially and relationally constructed and maintained, and its consequences both on sexual behaviours and the spread of HIV, both young men and women were included. A total of 16 young people 15–24 years old were interviewed, half of whom were women. Interviewees were recruited from the Salvation Army, Protestant and Revival churches.

### Interview topic guide

To explore the topic in depth and bring together the multiple views of the informants, we decided to use qualitative in-depth interviews (Kvale 1996). After being given an explanation of the purpose of the study and clarifications of any questions they might have had, participants were asked about previous or present school attendance, marital status and church affiliation (Table 1). After this introductory session, the first author went into issues regarding church messages on HIV prevention, gender, sexuality, health problems (if any) and life aspirations. The interviews were semi-structured based on a topic guide and with open-ended questions enabling H.L. to probe when necessary (Barbour & Schostak 2011). The interview topic guide facilitated conversations between the first author and informants and provided him access to the worlds of young churchgoers and their thoughts on masculinity and sexuality issues (Shenton & Hayter 2004).

**Table 1.** Basic demographics of the informants.

Participants in total (*n *= 16)	Women	Men
Mean age	20	19.6
*Churches*		
Protestant	4	4
Salvation Army	3	1
Revival churches	1	3
Students	6	7
Unemployed	2	1

### Data collection

For the interview, participants were given the freedom to choose to speak either Lingala, the vernacular language spoken in Kinshasa, or French. While some participants felt comfortable speaking in Lingala most opted for French, which is the official language in DRC. When asked about the reasons for their choice, some of them said that they felt more comfortable to address sexuality issues in French rather than in Lingala where sexual terms are perceived as offensive. The first author carried out all the interviews and made sure that the language utilised was suitable for the interviewees. The interviews lasted from 45 to 90 minutes and took place in locations where the youths felt comfortable speaking. These included their home or that of the first author, church buildings or under trees. We stopped after a total of 16 interviews when we had reached data saturation since further interviews would probably not have provided much new information in relation to the topics under investigation (Guest, Bunce & Johnson 2006). Data were collected from March through September 2010. Permission to record digitally the interviews was sought from the participants and all of them consented to this. The first author transcribed the recordings and translated them into English before analysis and discussions with the research team.

### Data analysis

**Fig. 1.**
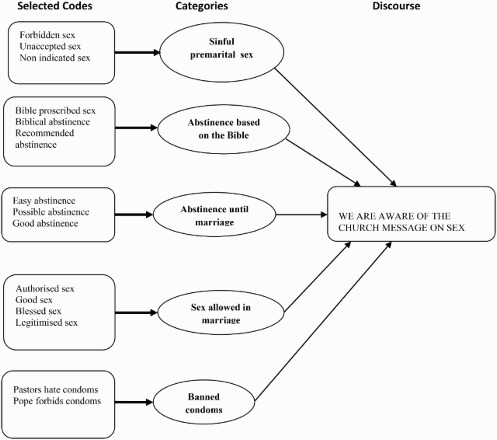
An example from the analytical process.

Data were analysed both manually and by using Open Code 3.2. The analysis was applied through the following five steps (Dahlgren, Emmelin & Winkvist 2007). The first step was the repeated reading of interview transcripts and listening to the interview recordings. This allowed the first author's immersion into the data. Secondly, a detailed line-by-line open coding was done to elicit important information from the interviews. The authors met regularly to review, discuss and reach agreement on similarities and differences in the meaning of codes. The third step was to identify important, relevant and emerging codes, to find axes between codes and to group them into categories. This process involved moving back and forward through the transcripts to analyse them iteratively as individual accounts and as a coherent whole. The fourth step attempted to find axes between the categories, and the final step was to identify pertinent discourses. An example of the analytical process is illustrated in Figure 1.

## Results

The five main discourses that emerged from the analysis were: We are aware of the church message on sex (Figure 1); Young men need sex; Young women need money; To use or not to use condoms and We trust in the church message. The different discourses are described below, and quotations are presented in italics.

### We are aware of the church message on sex

All participants said that they knew and listened to their church's message on sex based on the ideal morality of forbidden premarital sex, abstinence prescribed in the Bible, sex allowed in marriage and the banning of condoms. Young people were aware that premarital sex was perceived in the churches as harmful since it might entail the loss of virginity, unwanted pregnancies and illegal abortions. Sexual pleasure through premarital sex was considered dangerous because of the risk of HIV infection. Consequently, young people were taught to abstain from sex until marriage and, more importantly, to fear God by resisting sexual pleasure. Participants’ discourses about premarital sex were based on its sinful nature. ‘ … *It is true that the church does not accept sex before marriage; according to the church doctrine, premarital sex is a sin* … ’ (23-year-old man).

Also, interviewees stressed that church leaders expected older church youths to remain sexually abstinent until marriage, and thus serve as role models for younger peers. In this way, some participants stated that sexual abstinence was possible, and a good and realistic option that would spare youths from HIV and other sexually transmitted infections. While a few girls mentioned that virginity would please their husbands during their first sexual activities, nothing was said on boys’ chastity. Young women perceived boys to be knowledgeable about sex though they insisted that premarital sex was proscribed by the Bible. ‘ … *Young people should abstain from sex until marriage … according to the Bible principles … it is like that normally* … ’ (16-year-old woman).

Participants reported that their pastors prayed for and expected them to get married in the churches. That is why they have been taught to avoid casual sexual partners. Hence, sexual intercourse was perceived as sacred, good, accepted, blessed and legitimised, as long as it took place exclusively in marriage.

 … In the church we are told to protect ourselves from having sexual contact with men until marriage … this is the time one could have sex with a man … this is what we are told in the church … (24-year-old woman)

Interviewees noted that certain churches were silent over the issues of sex and sexuality probably because they lacked time to explore such complexities. In addition, pastors were said to be timid, ashamed and uncomfortable when talking about sex (the actual act) and sexuality (the cultural, social and psychological feelings related to sex), possibly because these deal with intimacy. Some participants said that a good pastor was not even expected to talk about sex and sexuality since such topics were perceived as inappropriate issues for the churches to handle.

 … Our pastors are busy with spiritual matters. They don't want to talk with us deeply about sexuality. They simply keep telling us to delay sex without showing us how sexual abstinence works in this broken world we live in … (24-year-old woman)

### Young men need sex

In spite of the existing ideal church-approved sexual morality that participants were aware of, some young men confirmed being sexually active and stated that sex was good and pleasurable. They desired sex, they initiated sex and determined its duration. Similarly, they heard but disobeyed the church message on abstinence since this was perceived as an old-fashioned message, and even seen as detrimental to their health:

 … (Laughing) … they want to ruin me. It would really destroy me. [ … ] I can see that this person does not like me because no one should be treated that way … Yes, the person who would tell me something like abstinence … you just can't do with anyone. I don't know who would bear sexual abstinence. Even Catholic priests can't cope with it and not even the nuns … (20-year-old man)

Many informants used metaphors for their sexual appetites such as young men are hungry, in a hurry and in need for premarital sex since their strong sexual needs would be uncontrollable. Young men also felt pressurised by their friends and older adults within their communities to start having sex before marriage to gain experience and to avoid being belittled by young women. In addition, they claimed that young women's provocative and sexy ways of dressing made young men unable to refrain from sex before marriage. Similar issues about what they perceived as sexual activities were also stressed among some young women:

 … Young men are thirsty for sex and are willing to do that … for them, they say that keeping … keeping sperm, it will hurt their back … .This is what young men say … keeping sperm will hurt my back, I have to release my sperm … (24-year-old woman)

According to the interviews, young male sexual behaviour was linked with their capacity to show sexual prowess and potency in sexual encounters, especially when they drink. They also reported that alcohol gave them energy to have sex with increased performance, which was also measured in terms of multiple ejaculations. They highlighted that the more a young man ejaculates, the stronger man he is sexually:

Some other men when they have sex with a woman … the woman should cry … in the meantime the man's sexual potency is still there and he is ready to have sex … (24-year-old man)

Exploring virgin girls and changing sexual tastes were raised as the main reasons for young men's engagement with multiple partners. Some young men said that sex with virgin girls was better than sex with sexually active girls, and in certain provinces the bride price for virgin girls was high. However, according to some participants, virginity was no longer of particular value in Kinshasa. Though young men were unemployed and lacked money, they reported that having sex with one partner was monotonous. Therefore, young men needed a variety of partners and considered themselves as sex players who could show young women that they had money in order to be considered as ‘real’ men:

 … A real man is someone who has a lot of money and many female partners. Yeah! These many female partners are provided with money in order to meet their needs. If they need something, the real man should buy it for them … (19-year-old man)

### Young women need money

The informants’ perception was that young women had sex with multiple partners for distinct but closely related reasons including love and money. Girls’ physical beauty and attractiveness were also perceived as commodities to trade with in order to acquire basic amenities such as food. In many cases, sex with multiple partners was a survival strategy in a context of economic hardship where parents were poor and uncaring. Certain female students were reported to have sex with multiple partners in order to pay school fees, obtain good grades in schools and acquire luxuries such as mobile phones and artificial hair. Participants also argued that even economically better-off women had many partners, not to exchange sex for money, but probably to maintain their socioeconomic status. However, women's financial dependency towards men was identified as the main reason for multiple partnerships:

One partner is not sufficient to give you all you need, not even parents … one guy will be responsible for providing body lotion and soap, and another will take care of transportation fees. Another one will care for clothes and shoes, and this is the way many young women behave (21-year-old woman)

Participants reported cases of girls who have sex with older men because young men are not capable of providing for them financially. While young women referred to older men as ‘fathers’ who follow little girls to have sex with them, young men also mentioned old women as ‘mothers’ who had sex with young boys partly because the latter were sexually stronger and more energetic. In both ways, adults provided financial support in exchange for sex. Participants said that older people were caring, kind and respectful towards their young partners. However, young women reported that older men requested unprotected sex because they paid for sex and were probably dissatisfied with sex while using condoms. Participants viewed age-disparate sex as unacceptable, and asserted that it could increase youth vulnerability to HIV and eventually lead to deadly sex. A couple of young women raised concerns about being in an unequal, age-disparate sexual relationship:

 … I have been dated and I am dating. I am using the two verbs because I have been dated by two married men. With both of them, when I consider their wives, I ask the question why these men can think of dating me, you see. When I see his wife I tell myself that she is a role model for me when I get married. But this man comes to me … and tells me the negative side of his wife … (21-year-old woman)

### To use or not to use condoms

Informants reported that some church leaders opposed both the health messages about condoms and the practice of condom use among churchgoers because this would motivate youths to have sex indiscriminately:

 … Condom protection? This is a church … Indeed, the pastor said that you should not say this here because we are in the church. If you start saying things like that in the church, this would promote sexual promiscuity. Indeed, young people would play with sex; therefore it is good if young people can abstain from sex … (22-year-old man)

In their sexual encounters, several respondents reported that youths did not seem to have much time for condom use; condoms were thought to be poorly manufactured and were sometimes unavailable. Overall, condoms were viewed as too tight, painful, delaying orgasm and having holes in them. According to some interviewees, condom use does not provide 100% protection in sexual encounters. Most participants noticed that young people favoured ‘natural’ sex because they perceive unprotected sex to be more pleasurable:

 … But the majority of young people say that having sexual intercourse with condoms is not pleasurable. Instead, they say if I have sex with my girlfriend, I would like to feel it … (23-year-old man)

By contrast, only a few youths mentioned using condoms in sexual encounters because they believed that condoms saved lives. Hence, they wished that church leaders would allow them to use condoms when necessary. Several participants reported that church leaders refusing the use of condoms could be suspected of being responsible for the spread of HIV among the youth. For these respondents, using condoms in this particular context of HIV was viewed as a sign of wisdom. Therefore, they disagreed with girls who refused to use condoms during sex. Such girls were viewed as untrustworthy, suspected to be pregnant or deemed to be already infected with HIV:

 … But if I happen to see a young woman who refuses to use condoms during sex, somebody like me, I will not accept having sex. I will refuse … Oh, pregnancy! I will be afraid of it during fertile days. However, I am first of all afraid of AIDS … (19-year-old man)

Similarly, some girls also showed agency in refusing unprotected sex in cases where partners did not use a condom. In such cases, boyfriends would be requested to buy one; otherwise sex would not take place. According to these girls, unprotected sex would only be appropriate with a sexual partner who had tested negative for HIV. ‘ … *This is my experience … if there is no condom … there is no sex* … ’ (21-year-old woman).

### We trust in the church message

A small group of church followers demonstrated their Christian values in two ways. Firstly, they recognised the church's moral ideal for sex as a viable option for themselves. Secondly, they perceived themselves as agents of change in promoting girls’ and boys’ equality in order to prevent HIV. Gender equality and mutual respect were mentioned as a prerequisite in order to build a gender-equitable society. For these church followers, boys’ superiority over girls was a meaningless issue. Instead, the respondents said that boys and girls should live together and share advice in order to seek true love. In sexual encounters, girls should be considered as equal decision-makers in initiating sex and enjoying sexual pleasure since girls have the same sexual desire as boys. ‘ … *In order to prevent HIV that we are talking about today, mutual respect is indeed very important* … ’ (21-year-old woman).

These church followers were aware that sexual abstinence was not an easy option and consequently used their prayers and faith in God to make sexual abstinence a reality. They insisted on continued adherence to church teaching to raise HIV awareness, and they trusted pastors who were telling them what they perceived to be the truth on sex, sexuality and HIV. ‘ … *As long as you hear the AIDS message in the churches, and you have confidence in the priest … if it is a priest that I trust, if that priest speaks about AIDS, I will protect myself* … ’ (18-year-old man).

Church followers also mentioned the increased risk of HIV that young people face in Kinshasa. This included peer pressure for an early sexual debut, intergenerational sex that can lead to death and people living with HIV who have unprotected sex. This group of young people perceived transactional sex and sex with a partner of unknown HIV status and the unwillingness of men to use condoms as potential HIV threats. They were fully aware of the existence of HIV in their midst and had therefore committed themselves to undergo voluntary HIV testing and counselling before marriage:

 … How can I marry a woman that I don't know the HIV status of? You always go to marry a young girl and you start sleeping with her. It is not worth it … You should finish your studies … you are responsible … once you see a woman, the first thing: HIV test. Both of you should take the test … (18-year-old man)

## Discussion

Discourses from the interviewed churchgoers revealed an obvious ambivalence between the church teachings of an ideal morality for sex and the ‘real world’ they live in with their sexual desire, pressure, expectations and experience. The churchgoers were heterogeneous in attitudes and sexual experiences. The majority were already sexually active given the gender norms coupled with the socioeconomic hardships of Kinshasa. While young men's discourses reflected sexual conquests to demonstrate their masculinities, young women's discourses referred to money and status acquired through transactional and intergenerational sex. These sexual relationships interfered with the church message related to forbidden premarital sex. A small number of youth committed themselves to believe in abstinence and to promote gender equality.

### Challenges of church morality for sex

The youths acknowledged the church teachings of sexual abstinence and faithfulness, which conflicted with premarital sexual activities that run counter to the teachings of sexuality in most Congolese churches. However, church leaders would probably gain a great deal by listening to the lived realities of young people's lives. Research has shown that HIV programmes involving young people and respecting their life patterns have a greater chance to succeed than programmes that do not prioritise such a holistic approach (UNAIDS 2003). Paradoxically, some respondents reported that many church leaders were reluctant to talk about sex and sexuality openly and truthfully. The lack of appropriate language regarding sexuality could partly explain the difficulties for church leaders to deliver effective messages about HIV prevention (Schmid 2005). Churches that view sex and sexuality negatively could be detrimental to young people, as this may encourage them to instead seek guidance from unqualified sources such as pornography (Paterson 2009).

The church banning of condoms may send a wrong signal that youths have the capacity to control their sexual lives through sexual abstinence they perceived as old fashion (Noden, Gomes & Ferreira 2010). We also found that condoms were banned on the grounds that they would legitimise young people's sexual promiscuity. By contrast, studies have shown that people exposed to appropriate information about sexuality tend to postpone their sexual activities (Boonstra 2011). The church anti-condom position could enable the spread of HIV among young people who have unprotected sex, thus rendering the church leadership a part of the HIV problem rather than a partner in its solution. This finding might challenge church leaders’ perceptions regarding youth sexuality to adjust rigid church doctrines to the realities of the young people. While the use of condoms might not be a perfect solution for all youths, the consistent and regular use of condoms might prevent HIV and save lives. In this way, the church leadership might make sure that sexually active youths willing to use condoms access them in order to protect themselves and others from HIV.

### Sexual conquest

Many participants noticed that the ‘real thing’ young men had to achieve in order to show their masculinities focused on penetration and ejaculation. Sexual strength was valued both as an indicator to sexually satisfy older women and a measure of sexual potency in relations with young women. Research has shown that young men may perceive sexual experience as a rite of passage into manhood. Additionally, young men can view sexual activities in terms of achievements rather than intimacy (Larkin, Andrews & Mitchell 2006) and consider girls’ bodies as sexual objects (Barker 2006). In our study, some young men were hardly concerned about the risks taken during unprotected sexual encounters. Instead, the primary ‘risk’ issue for them was failing to act as ‘a real man’. This type of widespread sexual stereotyping encourages pretence and should not go unchallenged (Clark 2011 [2009]).

Also, young men sought multiple and concurrent partners to showcase their masculinities. However, to maintain such a sexual network, they were expected to have money to distribute to young women. Most of the informants, however, were either students or unemployed (Table 1), as are the majority of youths in Kinshasa. Understanding these contradictions is crucial in order to ascertain what young men feel regarding the construction of masculine sexual behaviours and what it means to be a man. Young men need to learn that masculinity does not depend on sexual conquest and potency, but rather on the ability to behave responsibly and respectfully towards their partners (Barker, Verma, Crownover, Segundo, Fonseca, Contreras, *et al*., 2012).

### Transactional and intergenerational sex

We found that certain young women perceived sex as a commodity to trade for food. The same finding has been reported in another study where an association was found between women from poor family backgrounds and risk-taking behaviours to get money or food (Weiser, Leiter, Bangsberg, Buttler, Percy-de Korte, Hlanze, *et al*., 2007). Ignoring down-to-earth issues such as food could undermine effective HIV prevention strategies (Rollins 2007). The informants reported also that young women have many financial needs to be satisfied by multiple partners and interventions for HIV prevention have focused on females (UNFPA 2008). However, our study has revealed that young men also are involved in exchanging sex for money with older women. Although the HIV rates of young men involved in transactional sex are not well documented (Chatterji, Murray, London & Anglewicz 2005), sex with multiple partners can put women and men at high risk of HIV. Thus, interventions need to be adapted to address their respective specific needs.

Participants stressed that young women and men received kindness, care and luxury goods in age-disparate sex that they might miss with youths of their own age. Similarly, Masvawure (2010) stated that young women boosted their socioeconomic status through intergenerational relationships. However, in our study, young women felt bad about having sex with older men and probably thought that when they would get married, their husbands might also be having sex with young women. In a study where age and economic asymmetries were the norm between partners, Luke (2005) reported that older men were perceived to like sex while they hate to use condom because they pay young women for sex. Consequently, intergenerational relationships resulting into unsafe sex practices could partly explain the increased HIV prevalence among young women in the country (Leclerc-Madlala 2008).

### Church youth's contradictory discourses on gender

During the interviews several young men expressed respect for women and girls and affirmed that they would have mutual consenting and safe sex while at the same time reporting that ‘real’ men have unprotected sex with multiple partners. Our finding is consistent with the coexistence of gender-equitable and inequitable attitudes which has also been reported by Pulerwitz, Michaelis, Verma and Weiss (2010). Similarly, Torres, Goicolea, Edin and Öhman (2012) stated that the process of change regarding gender-equitable attitudes is complex and not linear. In their study, some young men showed more gender-equitable attitudes than others because of the support they received from significant others regarding the ongoing change; this was interpreted as a key strategy to challenge the rigid masculinity norms in the society.

### Alternative masculinities

Church followers who viewed themselves as agents of change to promote mutual respect and gender equality asserted, for instance, that young woman are also driven by the same feelings for sex as do young men (Oriel 2005). Therefore, church followers might represent the voices of resistance that challenge traditional norms of masculinities equating sexuality with masculinities (Barker 2005). In addition, church followers who chose to undergo HIV premarital test to know their HIV status and that of their partners might be considered as responsible people to be trained as peer educators to prevent HIV in their respective communities. This is a compelling evidence that gender is always relational and represent an entry point for church followers to deconstruct negative gendered behaviours in negotiation with each other (Connell & Messerschmidt 2005) and to construct positive ones to change and transform social norms of gender (Barker, Ricardo & Nascimento 2007). While Messerschmidt (2012) stated that women may cultivate hegemonic masculinities in romantic relationships, we found that young women advised their boyfriends to use condoms during sex. Otherwise sex would not take place. This is self-explanatory that young women may also help to (re)construct alternative masculinities.

### Study trustworthiness

The first author has been living and working in Kinshasa as a HIV church coordinator for some years and his pre-understanding of the local context might have affected his judgements and somehow influenced the answers he received from the participants. However, this was counteracted by having an open-mind attitude during the fieldwork and by the use of a prepared topic guide to keep the pre-understanding acknowledged and under control. Additionally, the first author informally discussed certain issues with young people to gain further clarification. The risk from ‘going native’ was further minimised by several discussions between the first author and the research team that was unfamiliar with the study setting (Lincoln & Guba 1985).

The interviews were rewarding because the first author had access to overlooked churchgoing young men and women whose sexuality is a topic that is both sensitive and reluctantly discussed in church settings in cross-gender and cross-age groups. However, care was taken not to be judgmental about young people's opinions during interviews. Moreover, the participants may have been influenced by social desirability since the first author was and is still working with churches to make them more competent regarding HIV. Some participants seemed to lack the language to address issues of sexuality. However, the participants’ freedom to express their opinions in the language in which they felt comfortable was helpful.

The results of our study are based on 3 specific communities out of 24 in Kinshasa, and the results may not reflect the discourse of all young churchgoers in this setting (Denscombe 1998). While the findings of this study should not be generalised to all churchgoers in the DRC, they provide invaluable insights on how church-going youths construct contradictory notions of gender and sexuality in this particular context of HIV.

## Conclusion

In the present study, we have identified five discourses and develop an image of their influence, affects and consequences on church youths. We found that young churchgoers are fully aware of the ideal sexual morality preached by their churches. However, this church teaching is not necessarily translated into expected actions. Instead, young churchgoers who are sexually active take risks by engaging in multiple relationships, which can include transactional and intergenerational sex. As a result, their sexual encounters are often unprotected, with sexual conquest being a key characteristic of masculinities for some participants. However, a few church followers made references to alternative masculinities, particularly with regard to some elements of church teachings such as gender equality and mutual respect between young women and young men. Although the process of changing gender identity is complex, alternative masculinities might promote gender-equitable attitudes especially among young men, and may open doors to young women and men to promote their respective well-being.
